# Metabolomics of laminae and midvein during leaf senescence and source–sink metabolite management in *Brassica napus* L. leaves

**DOI:** 10.1093/jxb/erx253

**Published:** 2017-09-06

**Authors:** Gilles Clément, Michaël Moison, Fabienne Soulay, Michèle Reisdorf-Cren, Céline Masclaux-Daubresse

**Affiliations:** INRA-AgroParisTech, Institut Jean-Pierre Bourgin, Saclay Plant Sciences, Versailles, France

**Keywords:** Leaf senescence, metabolomics, phloem, source–sink relationship

## Abstract

Leaf senescence is a long developmental process important for nutrient management and for source to sink remobilization. Constituents of the mesophyll cells are progressively degraded to provide nutrients to the rest of the plant. Up to now, studies on leaf senescence have not paid much attention to the role of the different leaf tissues. In the present study, we dissected leaf laminae from the midvein to perform metabolite profiling. The laminae mesophyll cells are the source of nutrients, and in C_3_ plants they contain Rubisco as the most important nitrogen storage pool. Veins, rich in vasculature, are the place where all the nutrients are translocated, and sometimes interconverted, before being exported through the phloem or the xylem. The different metabolic changes we observed in laminae and midvein with ageing support the idea that the senescence programme in these two tissues is different. Important accumulations of metabolites in the midvein suggest that nutrient translocations from source leaves to sinks are mainly controlled at this level. Carbon and nitrogen long-distance molecules such as fructose, glucose, aspartate, and asparagine were more abundant in the midvein than in laminae. In contrast, sucrose, glutamate, and aspartate were more abundant in laminae. The concentrations of tricarboxylic acid (TCA) compounds were also lower in the midvein than in laminae. Since nitrogen remobilization increased under low nitrate supply, plants were grown under two nitrate concentrations. The results revealed that the senescence-related differences were mostly similar under low and high nitrate conditions except for some pathways such as the TCA cycle.

## Teaser

Metabolomics of leaf laminae and veins during ageing reveal tissue specificities and different senescence programmes.

## Introduction

Oilseed rape (*Brassica napus* L.) is a major crop and the most widely cultivated oleaginous plant worldwide. Nitrogen fertilizer used to grow oilseed rape has dramatically increased over the last 40 years, due to the low nitrogen use efficiency (NUE) of this plant. Indeed, despite its strong capacity to absorb nitrate from the soil, oilseed rape displays the lowest NUE known in crops. This is due to the fact that older leaves drop and detach from the plant early and before nitrogen has been fully remobilized to the sink organs (Avice and Etienne, 2014; Girondé *et al.*, 2015). For oilseed rape, as for many other crops, nitrogen remobilization is a major determinant of the grain yield and of the seed quality, in terms of protein content (Zhao *et al.*, 2006; Chardon *et al.*, 2012) and certainly in terms of oil content, as suggested by the strong correlation found between nitrogen remobilization and seed carbon concentrations in Arabidopsis by Masclaux-Daubresse and Chardon (2011). Because of the early leaf drop, the loss of nitrogen was estimated to reach 100 kg N.ha^−1^.year^−1^. As oilseed rape cakes are used more and more to feed cattle, it also becomes important to increase protein contents in the seed remains after oil extraction. Thus improving nitrogen recycling and remobilization in oilseed rape in the early stages of the leaf senescence is of primary importance to maintain yield, reduce nitrate overuse, and improve the nutritional value of oilseed cake.

Leaf senescence is a long developmental process that is very important for plant physiology and metabolism. The leaf senescence phase starts when the leaf is fully expanded, and finishes when all the leaf tissues are dead. The n umerous molecular events occurring during leaf senescence are better known thanks to the many transcriptome analyses that have been done, essentially on Arabidopsis (Buchanan-Wollaston, 1997; Guo *et al.*, 2004), but also on oilseed rape (Buchanan-Wollaston and Ainsworth, 1997). These studies showed (i) that senescence events start before any leaf yellowing can be observed; and (ii) that as a step by step process, the main function of leaf senescence is the well-organized catabolism of all the cell constituents. It thus seems essential that cells stay alive as long as possible to manage efficient cellular nutrient recycling, phloem loading, and translocation to the seeds (Himelblau and Amasino, 2001; Diaz *et al.*, 2008). The methodical degradation of cell c onstituents starts with the chloroplast. As it is well known that the chloroplast is the main site for amino acid, lipid, and sugar synthesis, the consequences on the whole-leaf metabolome are important. Besides effects on chloroplast metabolism, the cell degradation processes orchestrated by autophagy, senescence- associated vacuoles, and proteases also produce new metabolites that can be used by the leaf itself or mobilized for translocation to plant sinks. The enzymes in charge of nutrient interconversions and metabolite transporters involved in the nutrient loading at the phloem level have been poorly studied (Masclaux-Daubresse *et al.*, 2008; Ludewig and Flügge, 2013). Watanabe *et al.* (2013) provided the most recent in-depth study of the metabolic changes occurring during leaf senescence in Arabidopsis. This study showed that the minor (low-abundance) amino acids such as γ-aminobutyric acid (GABA), branched chain amino acids (BCAAs), and aromatic amino acids (AAAs) accumulated during leaf senescence while the most abundant (glutamate, aspartate, asparagine, glutamine, and serine, all referred to as major amino acids) decreased. The accumulation of minor carbohydrates (minor CHOs) as cell wall CHOs, low-abundance s ugars and alcohols, and free fatty acids was also shown. While most of the changes described in Arabidopsis senescent leaves by Watanabe *et al.* (2013) had been also found by Diaz *et al.* (2005) in five different recombinant inbred lines grown under nitrate deficiency, it is not known whether these changes were specific for the Arabidopsis model plant and whether nitrate-dependent modifications could be different in other species. In addition, it has never been investigated whether metabolite changes with ageing could be different depending on the leaf tissues and especially in the vein compared with the mesophyll and parenchyma of laminae.

In this study, we compared the metabolic changes occurring in oilseed rape leaves during senescence. Both nitrate-sufficient and nitrate-limited plants were grown, knowing that nitrate limitation induces nitrogen remobilization and leaf senescence. We dissected leaves, considering laminae and the primary vein separately in order to compare metabolite contents in these two different tissues. The results showed that for most of the metabolites, their concentrations are modified during ageing, and sometimes in different ways in midvein and laminae tissues. The high hexose and amide concentrations found in the midvein compared with laminae suggest different senescence programmes, and an important role for the veins in the metabolic changes occurring in leaves during senescence.

## Materials and methods

### Plant material and growth conditions

*Brassica napus* L. plants of the Darmor-bzh genotype were grown in a greenhouse at INRA Versailles, France. Seeds were sown on sand and watered with nutrient solution for 2 weeks in order to allow germination and subsequent growth of plantlets. When the first two true leaves appeared, plantlets were transferred into pots containing sand and were separated into two groups with contrasting nitrogen fertilization regimes: (i) HN for high nitrate [3 mM KNO_3_, 2.5 mM Ca(NO_3_)_2_, 1 mM MgSO_4_, 0.5 mM KH_2_PO_4_, 30 µM H_3_BO_3_, 27 µM FeNa-EDTA, 10 µM MnSO_4_, 1 µM ZnSO_4_, 1 µM Na_2_MoO_4_, 0.5 mM CuSO_4_, and 0.5 µM Co(NO_3_)_2_] and (ii) LN for low nitrate [0.4 mM KNO_3_, 3 mM KCl, 2.5 mM CaCl_2_, 1 mM MgSO_4_, 0.5 mM KH_2_PO_4_, and the same micronutrients as those in HN solution] (Albert *et al.*, 2012). At 56 d after sowing, four plants of each nutrition regime were harvested and sampled. For each plant, all leaf ranks were collected. Each leaf was quickly weighted and dissected in order to separate the petiole plus primary vein from the rest of the leaf. This constituted two samples per leaf, referred to herein as the vein (for petiole plus primary vein) and the laminae (for the deveined leaf blade). All fresh samples were chilled immediately in liquid nitrogen and stored at –80 °C.

### Chlorophyll determination

Chlorophyll content was determined using the SPAD-502 chlorophyll meter (Konica-Minolta, Carrière sur Seine, France).

### Metabolite profiling using GC-MS

Extraction, derivatization, analysis, and data processing were performed according to Fiehn (2006). The ground frozen samples (20 mg FW) were resuspended in 1 ml of frozen (–20 °C) water:acetonitrile:isopropanol (2:3:3) containing ribitol at 4 µg.ml^–1^ and extracted for 10 min at 4 °C with shaking at 1400 rpm in an Eppendorf thermomixer. Insoluble material was removed by centrifugation at 20 000 *g* for 5 min. A 100 µl aliquot of supernatant was collected and dried for 4 h in a Speed-Vac. The whole series (94 samples) was stored at –80 °C, and samples were analysed in four subseries and redried for 2 h before derivatization. Three blank tubes underwent the same steps as the samples. After drying, 10 µl of 20 mg ml^–1^ methoxyamine in pyridine were added to the samples. The reaction was performed for 90 min at 28 °C under continuous shaking in an Eppendorf thermomixer. A 90 µl aliquot of *N*-methyl-*N*-trimethylsilyl-trifluoroacetamide (MSTFA) (Aldrich, Saint Quentin Fallavier, France; Product number 394866, 10 × 1 ml) was then added and the reaction continued for 30 min at 37 °C. After cooling, 45 µl of the derivatized sample were transferred to an Agilent (Les Ulis, France) vial for injection.

Metabolites were analysed by GC-MS 4 h after derivatization. A 1 μl aliquot of the derivatized samples was injected in splitless mode on an Agilent 7890A gas chromatograph coupled to an Agilent 5975C mass spectrometer. The column was an Rtx-5SilMS from Restek (30 m with a 10 m Integra-Guard column). The liner (Restek 20994) was changed before each series of analyses and 10 cm of column was cut. The oven temperature ramp was 70 °C for 7 min then 10 °C min^–1^ to 325 °C for 4 min (run length 36.5 min). Helium constant flow was 1.5231 ml min^–1^. Temperatures were as follows: injector, 250 °C; transfer line, 290 °C; source: 250 °C; and quadripole, 150 °C. Samples and blanks were randomized. Amino acid standards were injected at the beginning and end of the analysis to monitor the derivatization stability. An alkane mix (C10, C12, C15, C19, C22, C28, C32, and C36) was injected in the middle of the queue for external calibration. Five scans per second were acquired. Metabolites, analysed using a GC-MS technique, were annotated and their levels on a fresh weight basis were normalized with respect to the ribitol internal standard.

### Metabolomic data processing

Raw Agilent datafiles were converted to NetCDF format and analysed with AMDIS (http://chemdata.nist.gov/dokuwiki/doku.php?id=chemdata:amdis). A home retention index/mass spectra library, built from the NIST, Golm, and Fiehn databases and standard compounds, was used for metabolite identification. Peak areas were then integrated using the QuanLynx software (Waters, Guyancourt, France) after conversion of the NetCDF file to MassLynx format. Statistical analysis was done with TMEV (http://mev.tm4.org/#/welcome); univariate analysis by permutation (one- and two-way ANOVA) was first used to select the significant m etabolites. Multivariate analysis [hierarchical clustering and principal component analysis (PCA)] was then carried out in order to establish the metabolite clusters. Only metabolites showing repeatable and significant differences (based on a *t*-test) according to leaf age and nitrate growth conditions are presented. Absolute quantification was performed by a one-point calibration in splitless mode and another one-point calibration in split mode, using a 4 ng injection of external standards.

## Results

### Characterization of metabolic changes associated with leaf senescence in oilseed rape in relation to nitrate nutrition

The leaf material used for the metabolomic analyses was the same as used by Orsel *et al.* (2014) to investigate the *GS1* glutamine synthetase gene expression levels. The GS1-coding genes are known as senescence-related genes in many plant species. Therefore, results from Orsel *et al.* (2014) provided a good basis that characterizes the leaf senescence gradient in the oilseed rape leaves studied here. It should be noted that the metabolomic data presented here have not been published previously by Orsel *et al.* (2014).

**Fig. 1.**
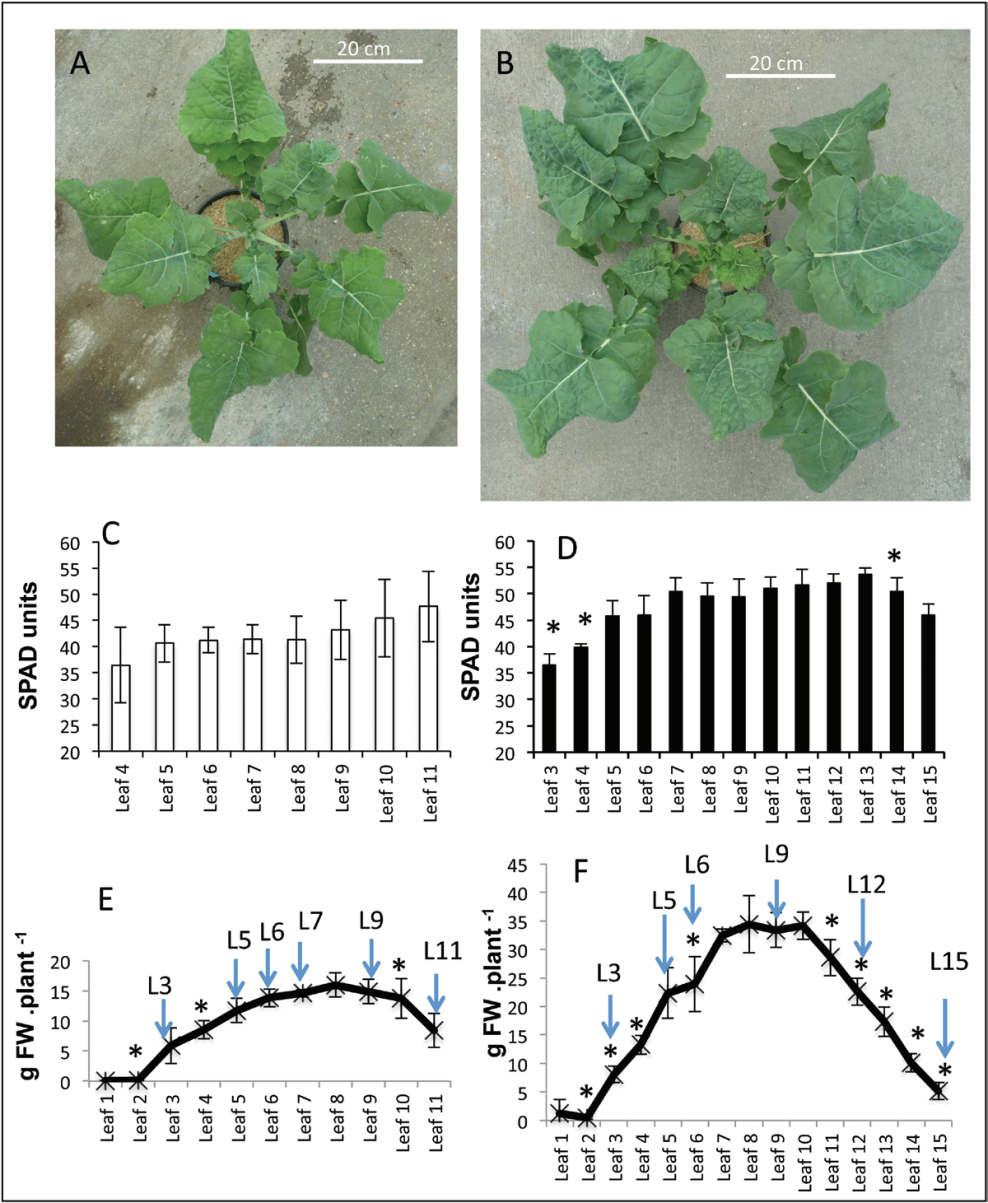
Natural leaf senescence in oilseed rape. Leaf senescence was monitored in leaves of plants grown under low (LN; A, C, E) and high (HN; B, D, F) nitrate conditions for 56 d after sowing. Leaf ranks were numbered from the taproot to the apex. Leaf 1 (L1) is the oldest leaf. Leaves 11 and 15 (L11 and L15) were the youngest leaves harvested from the LN and HN plants, respectively. Changes in chlorophyll contents were measured by SPAD (C, D). Leaf (laminae plus main vein) fresh weight was lower in LN plants (E) than in HN plants (F). All data represent the mean ± SD of four biological replicates. * indicates significant difference between leaf *n* and leaf *n*+1 (*t*-test *P*<0.05). Leaves harvested and used further for metabolite profiling are indicated by arrows.

Leaves were harvested from plants grown under low (LN) or high (HN) nitrate conditions. A total of 11 leaves were harvested on the LN-grown plants and 15 on the HN-grown plants. Leaves were numbered from 1 to 11 and 1 to 15 from the bottom to the top of the LN and HN plants, respectively (Fig. 1). For the same leaf number, leaf biomass was twice as high in HN plants than in LN plants. Chlorophyll concentrations estimated using SPAD units did not reveal a strong decrease with ageing, although a tendency can be observed and significant decreases from leaf *n* to leaf *n*+1 were estimated under HN (Fig. 1). However, leaf biomass and *GS1* and *GS2* expression levels (Supplementary Fig. S1 at *JXB* online) monitored by Orsel *et al.* (2014) were a good basis from which to choose six leaf ranks (shown with arrows on Fig. 1E and F) to investigate the metabolic changes occurring with leaf ageing under LN and HN. The main vein (including the petiole) and the laminae were separated to monitor and compare senescence-associated changes in these tissues. The metabolites clearly identified and quantified using GC-MS analyses are listed in Supplementary Data Set S1. One-way ANOVA was performed in order to identify metabolites significantly modified in the different samples. Significant metabolites were used to perform hierarchical clustering analysis. The first level of clustering separated organs, the second level separated nitrate nutrition, and the third level revealed the senescence effect (Supplementary Data Set S2). These clusterings were clearly revealed by PCA as shown in Supplementary Fig. S2.

From the hierarchical clustering presented in Supplementary Data Set S2, we extracted significant metabolites and removed unknown metabolites. For a better reading, Supplementary Data Set S3 presents the mean and SD of the 106 significant and identified metabolites selected, and their clusters on the basis of their relative senescence- and nitrogen-related changes. Class 1 contains 60 metabolites that decreased with leaf ageing (Down during senescence). Class 2 contains 12 metabolites that increased with ageing (Up during senescence). Class 1 and Class 2 were then subdivided depending on the metabolite concentrations under LN and HN and depending on the metabolites found in the midvein and laminae. Globally, the senescence-related trends observed (Up or Down during senescence) were the same in laminae and in the midvein (Supplementary Fig. S3). The other metabolites, without clear up- or down-regulation during ageing, were in classes 3–10 of the Supplementary Data Set S3.

**Fig. 2.**
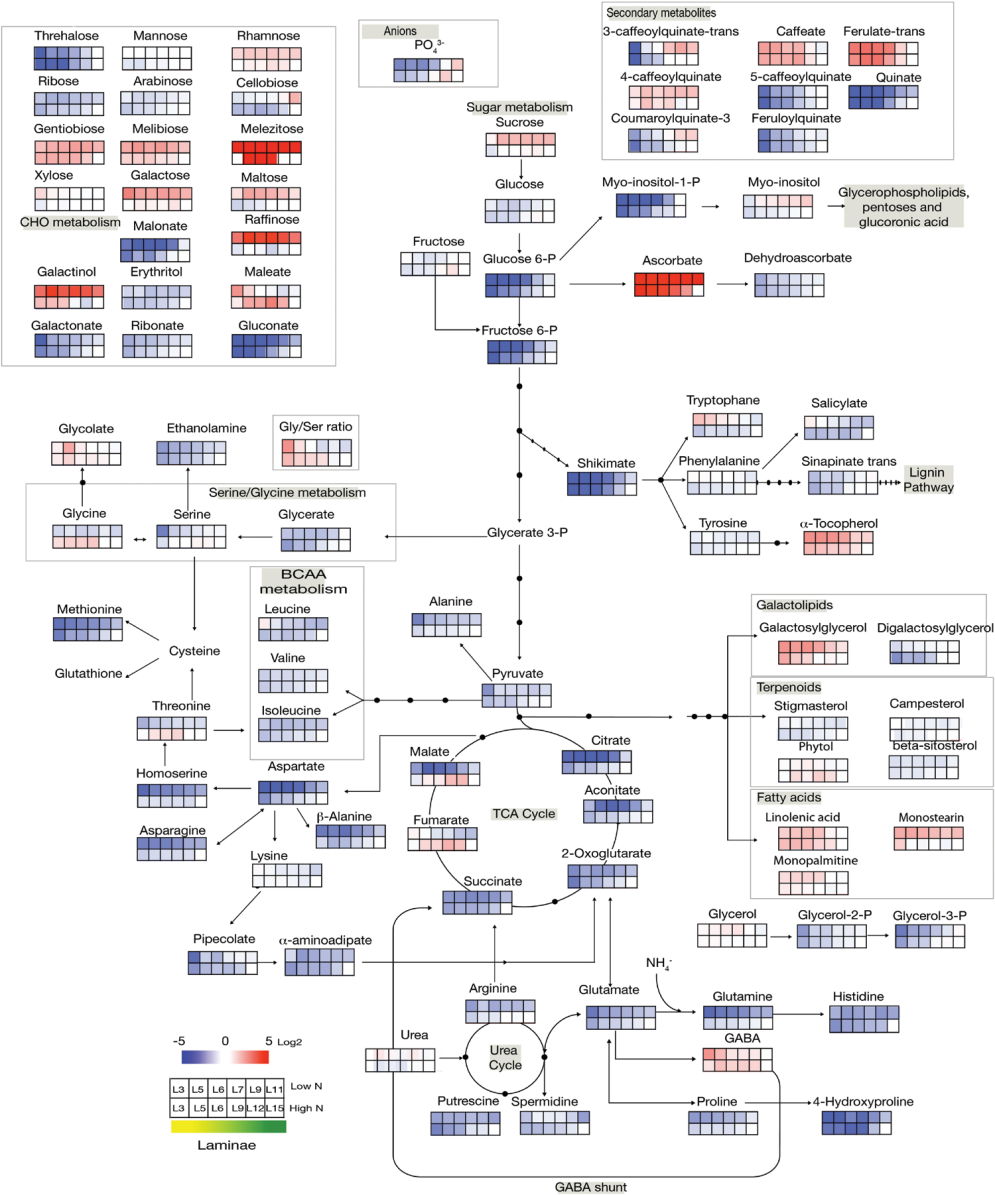
Heat map of metabolite changes in the laminae of oilseed rape leaves during senescence. Leaf laminae were harvested from plants grown under low (LN) or high (HN) nitrate conditions. Metabolite concentrations were determined as the peak area in GC-MS analyses normalized to sample fresh weight. Log_2_ of the ratios of the metabolite concentrations to their value in the youngest leaf of the high nitrate treatment (HNL15) are presented on a metabolic pathway representation by shades of red or blue colours according to the scale bar. The stage of senescence of each leaf rank is indicated by shades of colours from yellow (more senescent old leaf) to green (less senescent young leaf) according to the scale bar. Data represent mean values of four biological replicates for each leaf rank and time point. L3 to L11 or L15, laminae of leaf ranks from the oldest (L3) to the youngest L11 and L15 for LN- and HN-grown plants, respectively.

**Fig. 3.**
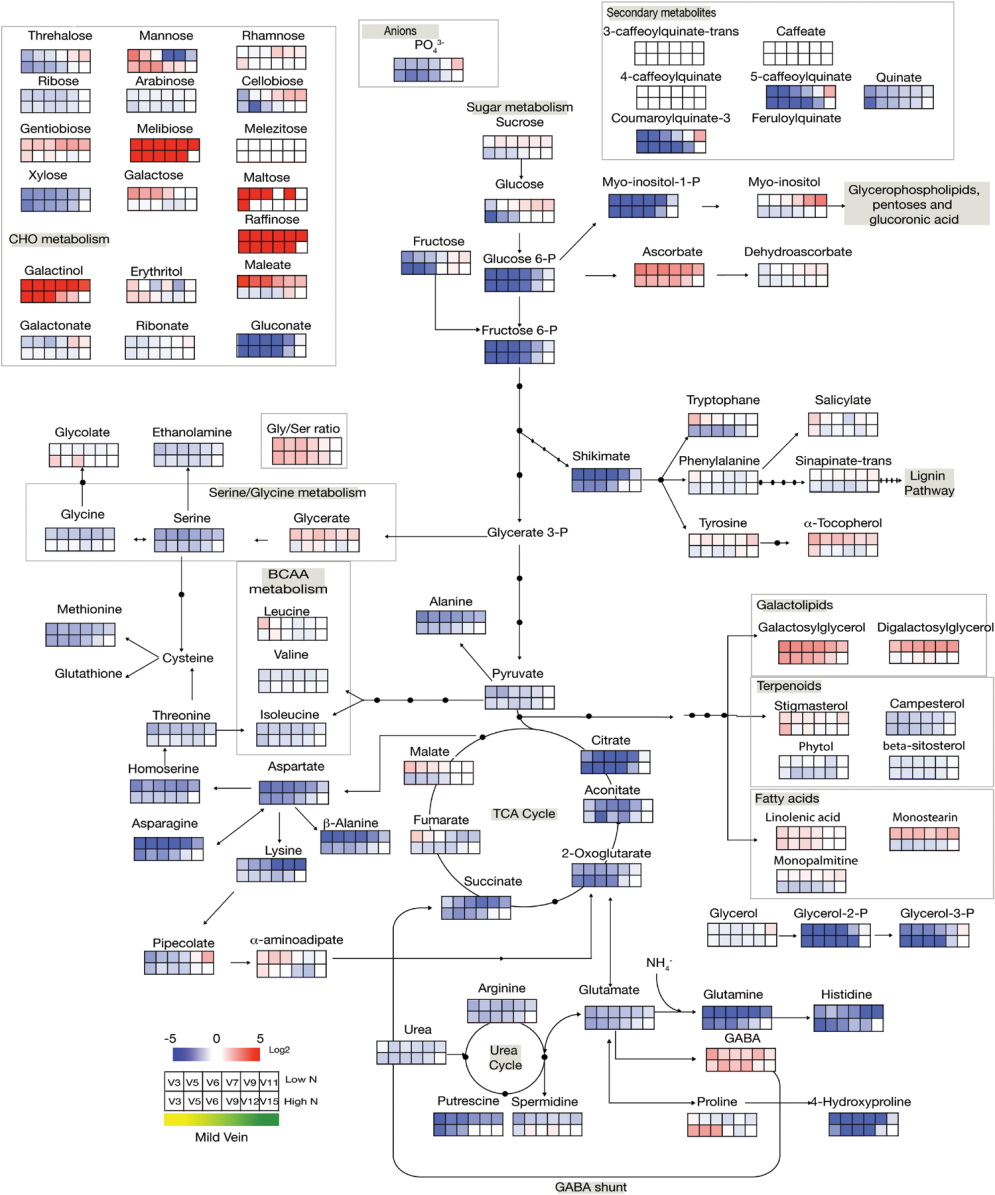
Heat map of metabolite changes in the midvein of oilseed rape leaves during senescence. The main vein of leaves was harvested from plants grown under low (LN) or high (HN) nitrate conditions. Metabolite concentrations were determined as the peak area in GC-MS analyses normalized to sample fresh weight. Log_2_ of the ratios of the metabolite concentrations to their value in the youngest leaf of the high nitrate treatment (HNV15) are presented on a metabolic pathway representation by shades of red or blue colours according to the scale bar. The stage of senescence of each leaf rank is indicated by shades of colours from yellow (more senescent old leaf) to green (less senescent young leaf) according to the scale bar. Data represent mean values of four biological replicates for each leaf rank and time point. V3 to V11 or V15, midvein harvested from the different leaf ranks, from the oldest (V3) to the youngest V11 and V15 for LN- and HN-grown plants, respectively.

The senescence-related and nitrogen-related changes were then examined after normalizing all the metabolite concentrations in the midvein and laminae to their respective concentrations in the youngest leaf [i.e. leaf number 15 of HN plants (HNV15 and HNL15), and leaf number 11 of LN plants (LNV11 and LNL11), respectively]. The log_2_ ratios were computed and the relative metabolite concentrations were presented on metabolic maps (Fig. 2 for the laminae and Fig. 3 for the midvein) using false colours. Results in Figs. 2 and 3 show that globally the age-related changes were similar under LN and HN conditions for the majority of the metabolites. The resemblance between Figs 2 and 3 also shows that globally the age-related changes in the laminae and in the midvein were similar (Supplementary Fig. S3). However, s everal exceptions could be identified and are detailed below.

### Leaf ageing hallmarks and specific metabolite changes in leaf laminae and midvein under both LN and HN conditions

The senescence effect was globally similar in laminae and midvein (Figs 2, 3; Supplementary Fig. S3). Almost all the amino acids decreased with ageing except GABA and lysine, which increased. Glycolysis sugars decreased with ageing, while most of the minor CHOs and lipids accumulated.

There were, however, some more specific changes that depended on either nitrate concentration or tissues. For example, changes in glycine, threonine, and tryptophan in laminae were opposite depending on the nitrate conditions. Proline decreased with ageing in laminae independently of nitrate availability, but increased in the midvein only under HN conditions. α-Aminoadipate, which is a lysine degradation product, increased with ageing only in the midvein of LN plants.

The mannose, xylose, and erythritol minor CHOs were differentially modified by ageing in laminae and midvein. Maltose and xylose were differentially affected depending on nitrate conditions. Also digalactosylglycerol, stigmasterol, and phytol changes were different in midvein and laminae, possibly due to the higher chloroplast abundance in laminae.

**Fig. 4.**
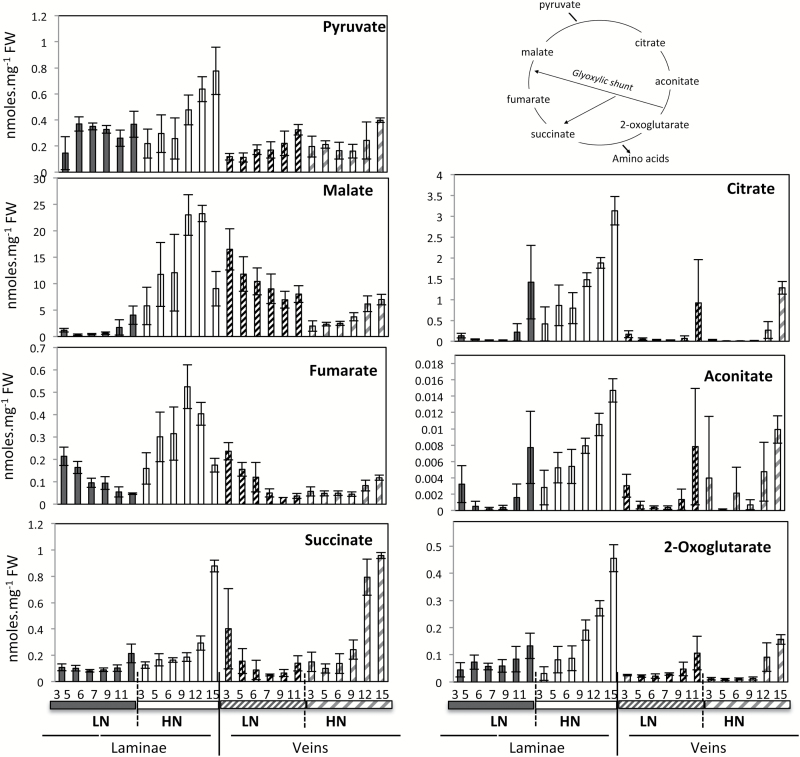
TCA changes during leaf senescence in oilseed rape. Laminae (on the left) and midvein (on the right) of plants grown under LN and HN conditions were analysed. Metabolite concentrations were determined by GC-MS analyses and normalized to sample fresh weight as nmol.mg^–1^ FW. Data represent mean values and SDs of four biological replicates for each leaf rank and time point.

Changes in tricarboxylic acid (TCA) compounds were more complex especially under LN (Figs 2, 3). In HN plants, all the TCA compounds (citrate, aconitate, 2-oxoglutarate, malate, and fumarate) decreased with ageing in both laminae and midvein (Fig. 4). In LN plants, citrate, aconitate, and 2-oxoglutarate decreased with ageing in both laminae and midvein. The trend was different for succinate, malate, and fumarate. Succinate and malate increased with ageing in the midvein, and fumarate increased with ageing in both laminae and midvein of LN plants (Fig. 4). The results thus reveal major discrepancies in the senescence effect on fumarate, malate, and succinate concentrations when nitrate is limiting, and especially in vein tissues (Fig. 4). This could be due to a different activity in the glyoxylic shunt under HN and LN.

### Metabolite relative concentrations in laminae and midvein reveal tissue specializations

The use of external standards and the comparison of the GC-MS results with anion-exchange/ninhydrin-detected concentrations of several sets of samples (unpublished results) allowed us to transform the metabolite peak area per mg FW into nanomoles per mg FW.

Interestingly, we could then observe that the concentrations and the distributions of several metabolites were very different in the midvein and in the laminae (Supplementary Fig. S4). This suggested a specialization of the leaf tissues in either metabolite synthesis or metabolite transport and xylem or phloem loadings.

**Fig. 5.**
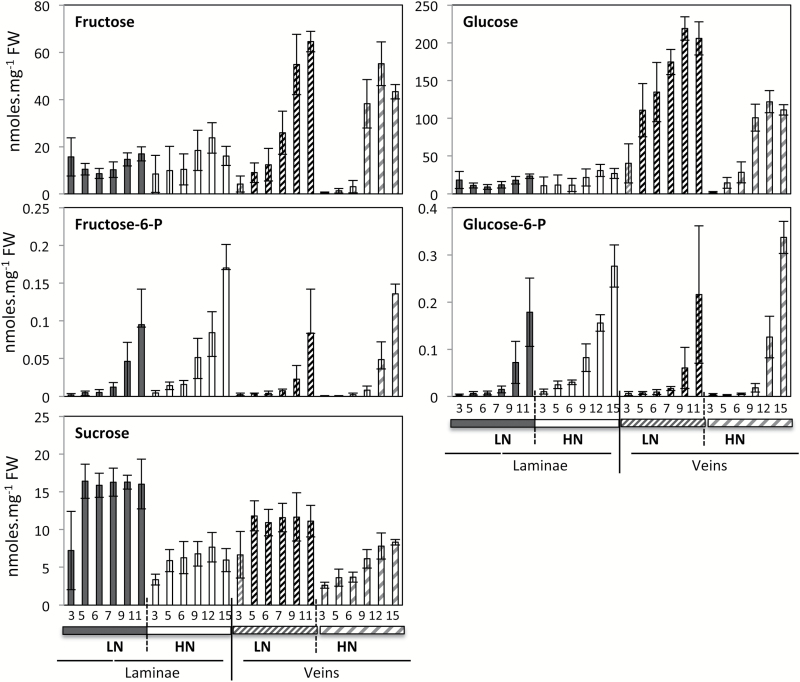
Sugar decreases during senescence are different in laminae and midvein of oilseed rape leaves. Changes were monitored during leaf senescence in oilseed rape laminae (on the left) and the midvein (on the right) of plants grown under LN and HN conditions. Metabolite concentrations were determined by GC-MS analyses and normalized to sample fresh weight as nmol.mg^–1^ FW. Data represent mean values and SDs of four biological replicates for each leaf rank and time point.

Glucose and fructose were much more abundant in the midvein than in the laminae. Their decrease with ageing thus appeared much more severe in the midvein (Fig. 5). In contrast, sucrose, glucose-6-phosphate, and fructose-6-phosphate concentrations were not different in the two tissues, underlining the fact that midveins are actually glucose- and fructose-rich tissues.

**Fig. 6.**
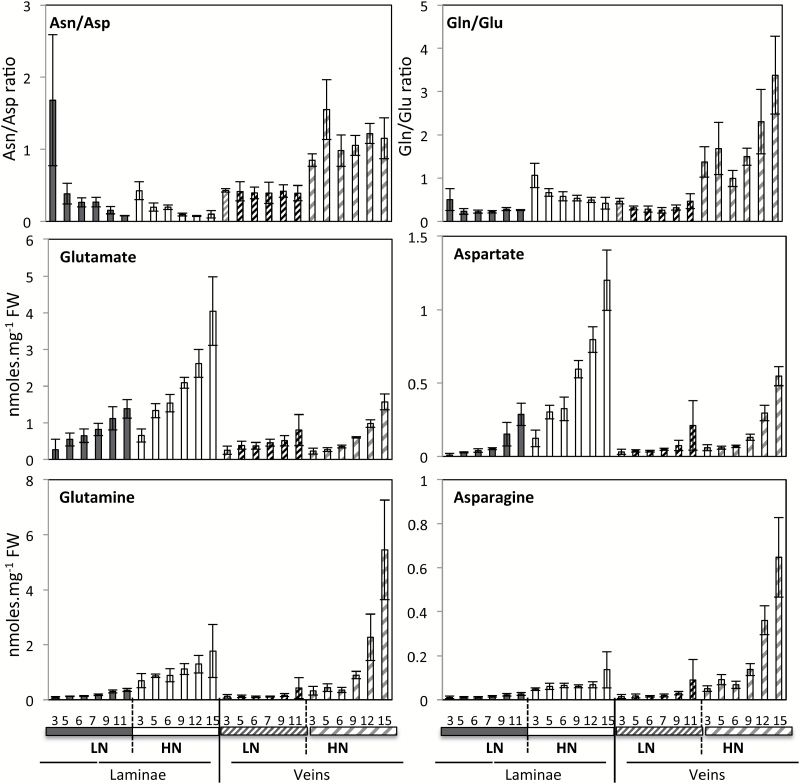
Glutamate, aspartate, glutamine, and asparagine decrease during senescence in laminae and midvein of oilseed rape leaves. Changes were monitored during leaf senescence in oilseed rape laminae (on the left) and midvein (on the right) of plants grown under LN and HN conditions. Metabolite concentrations were determined by GC-MS analyses and normalized to sample fresh weight as nmol.mg^–1^ FW. Glutamine/glutamate and asparagine/aspartate ratios are presented. Data represent mean values and SDs of four biological replicates for each leaf rank and time point.

The comparison of the distributions of glutamate, glutamine, aspartate, and asparagine in the midvein and laminae also exhibited clear differences. Glutamate and aspartate were more abundant in the laminae, while glutamine and asparagine accumulated in the primary vein (Fig. 6). Although glutamate, glutamine, aspartate, and asparagine decreased with leaf ageing in both laminae and midvein, the asparagine/aspartate and glutamine/glutamate ratios changed in opposite ways in the two tissues. Ratios increased in laminae and decreased in midvein with ageing, revealing different interconversion levels in the two tissues depending on the leaf age (Fig. 6). This suggested that both glutamine and asparagine synthesis in laminae and midvein, and translocation to the veins and into the phloem saps control source–sink relationships. Proline is a direct product of glutamate and its catabolism directly resupplies glutamate, thus conferring proline a role in buffering glutamate contents in plants. Interestingly, proline concentrations increased with ageing in the midvein of the HN leaves and decreased in the laminae of the same leaves. This suggested that the translocation from laminae to midvein could control relative proline contents in the two tissues. While the magnitudes of the proline concentrations in midvein and laminae were not very different, the opposite effect of senescence on proline concentrations in the laminae and midvein suggested that proline is especially dedicated to phloem loading and export from the source leaves (Figs 2, 3).

### Low nitrate markers in oilseed rape

**Fig. 7.**
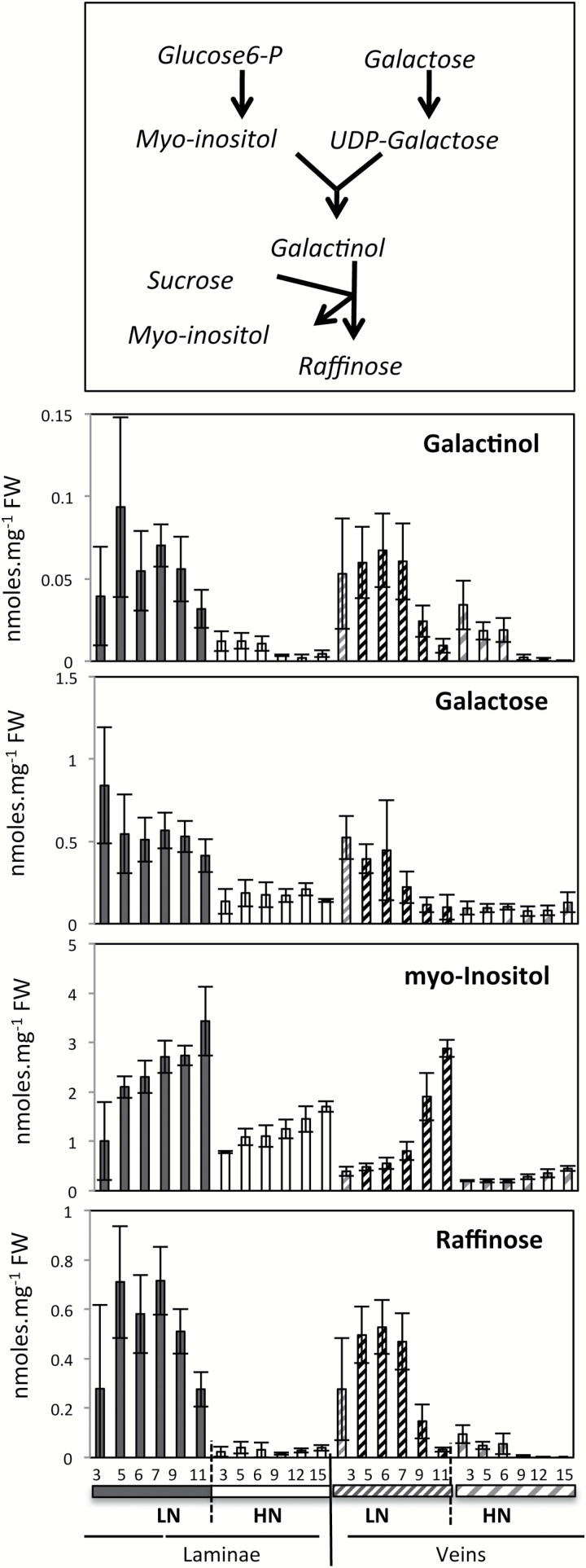
Galactinol, galactose, myo-inositol, and raffinose are more abundant in LN oilseed rape laminae and are differentially modified during leaf senescence. Changes were monitored during leaf senescence in oilseed rape laminae (on the left) and midvein (on the right) of plants grown under LN and HN conditions. Metabolite concentrations were determined by GC-MS analyses and normalized to sample fresh weight as nmol.mg^–1^ FW. Data represent mean values and SDs of four biological replicates for each leaf rank and time point.

A few metabolites changed in different ways depending on the nitrate conditions. They can be considered as nitrate response markers. For example, sucrose, galactinol, galactose, and raffinose accumulated to a much greater extent in the leaves under LN conditions relative to HN (Fig. 7). Because they were more abundant under LN, galactinol, galactose, and raffinose presented sharper senescence-related increases under these conditions. Myo-inositol, which is a substrate of galactinol and a co-product of raffinose biosynthesis, was accordingly more abundant under LN. However, it was surprisingly inversely regulated by ageing, accumulating to a high level preferentially in the younger leaves (Fig. 7). Finally it is striking that most of the amino acids (Figs 2, 3) and especially glutamate, aspartate, glutamine, and asparagine (Fig. 6) are more abundant under HN than under LN. Glutamine showed remarkably high concentrations in veins of the youngest leaves harvested from HN plants as compared with those from LN plants.

### Redox homeostasis and leaf senescence in oilseed rape

Ascorbate/dehydroascorbate and α-tocopherol are markers of oxidative stress in plants. The large increase in α-tocopherol and ascorbate and the decrease of dehydroascorbate with ageing revealed higher antioxidant activities in old oilseed rape leaves (Figs 2, 3; Supplementary Fig. S3). Ascorbate concentrations were quite similar in the two tissues, whereas dehydroascorbate was much higher in laminae than in midvein (Supplementary Data Set S3). However, the fact that the ascorbate concentration increased while dehydroascorbate decreased with ageing was consistent with the interconversion of these two metabolites. The higher concentration of dehydroascorbate in laminae could be related to the higher oxidative stress related to the photosynthetic activity in this tissue.

## Discussion

After tobacco and soybean, Arabidopsis was adopted as the main plant model for leaf senescence studies, and it has been used for the development of most of the molecular senescence markers (Buchanan-Wollaston, 1997; Buchanan-Wollaston *et al.*, 2003; Gepstein *et al.*, 2003; Guo *et al.*, 2004). In 2000, Masclaux *et al.* published metabolite profiling of tobacco leaves to characterize senescence modifications. The authors showed transient sugar and starch accumulations in mature leaves followed by a decrease in the senescing leaves. They revealed the accumulation of GABA with ageing and confirmed the very high contents of proline in the y oungest leaves (up to 20%), and its sharp decrease with ageing (Mizuski *et al.*, 1964). Diaz *et al.* (2005) showed quite similar senescence features in Arabidopsis plants grown under LN conditions. Sugar and starch transiently accumulated d uring leaf expansion and decreased with senescence. Aspartate, asparagine, and glutamine decreased steadily, and glutamate transiently increased in mature leaves before decreasing in senescing leaves in the same manner as sugars. In contrast, the minor amino acids (leucine, isoleucine, arginine, t yrosine, and, more importantly, GABA) increased with ageing and especially during the senescence phase, probably due to their release from protein degradation by proteases. Further, Watanabe *et al.* (2013) gave a much wider picture of ageing effects using new -omics tools. The metabolic changes in Arabidopsis published by Diaz *et al.* (2005) were then confirmed by GC-MS and, in addition, they showed that minor CHOs and lipids accumulated with ageing. More recently, Chrobok *et al.* (2016) confirmed that most of the major amino acids decreased with senescence in Arabidopsis, while the BCAAs and AAAs remained quite high in senescing leaves. In barley, Avila-Ospina *et al.* (2017) depicted similar metabolite changes during leaf senescence. The authors showed the decrease of sugar and major amino acid contents with ageing and the accumulation of lipids, CHOs, and minor amino acids (leucine, tyrosine, and tryptophan) in barley. They also revealed that changes in TCA compounds were more complex and different depending on the nature of the leaves considered (primary or flag leaves) and on the plant nutrition.

In the present study, we show that the global leaf senescence features described in previous studies are conserved in oilseed rape. As in other plants, leaf ageing in oilseed rape is paralleled by (i) the decrease of sugars; (ii) the decrease of the major amino acids such as glutamate, aspartate, asparagine, and glutamine; and (iii) the increase of fatty acids and CHOs. This study thus provides additional evidence that such a global picture of senescence-related leaf metabolite changes is common to all the species studied so far. As shown in other dicotyledonous plants (Arabidopsis and tobacco), GABA accumulated in the oilseed rape leaves with senescence. GABA can be confirmed as one of the best senescence markers in tobacco, Arabidopsis, and oilseed rape (Masclaux *et al.*, 2000; Diaz *et al.*, 2005; Watanabe *et al.*, 2013) and thus, regarding a barley study (Avila-Ospina *et al.*, 2017), it could be a dicot-specific senescence marker. Directly synthesized from glutamate, GABA provides succinate, through the GABA shunt, to fulfil the TCA cycle. This is one of the anaplerotic pathway described in plants (Shimajiri *et al.*, 2013; Xiong *et al.*, 2014). Lysine is involved in another anaplerotic pathway that involves pipecolate and α-aminoadipate and provides 2-oxoglutarate (Boex-Fontvieille *et al.*, 2013). Lysine increased with ageing under LN conditions, but pipecolate and α-aminoadipate decreased in parallel, thus suggesting that lysine catabolism was a checkpoint controlling the fluxes to TCA replenishment. Interestingly, GABA and lysine accumulated in a similar manner during senescence in oilseed rape and Arabidopsis leaves (Araújo *et al.*, 2010, 2011; Chrobok *et al.*, 2016).

Unlike in Arabidopsis or tobacco, there was no accumulation of any BCAAs (valine, leucine, and isoleucine) or AAAs (tryptophan, phenylalanine, and tyrosine) in the s enescing oilseed rape leaves. This suggested that the BCAAs and AAAs released from protein catabolism were used for nitrogen remobilization or to support mitochondrial respiration in oilseed rape senescing leaves (Araújo *et al.*, 2010). The reason why BCAAs and AAAs accumulated in Arabidopsis leaves but not in oilseed rape leaves during leaf senescence could be that nitrogen remobilization is more efficient in the oilseed rape crop, bred for this trait, than in the wild Arabidopsis plant. Considering remobilization processes, it can indeed be seen that urea, which is released from amino acid catabolism (Bohner *et al.*, 2015), accumulated in the senescing Arabidopsis rosette leaves (Watanabe *et al.*, 2013) but decreased in the oilseed rape leaves with ageing, thus supporting the hypothesis of a better nitrogen remobilization in oilseed rape. The absence of BCAA and AAA accumulation in old leaves of oilseed rape could also be due to differences in the selective remobilization of minor amino acids in Arabidopsis and *B. napus*. Selective amino acid translocations, from source to sink through the phloem or the xylem, have indeed been demonstrated by several groups in the 1980s, although mainly in legume species (McNeil *et al.*, 1979; Murray, 1992).

Nitrogen remobilization is usually associated with the synthesis of glutamine and asparagine by asparagine synthetase (AS) and cytosolic glutamine synthetase (GS1) (Masclaux-Daubresse *et al.*, 2008). Accordingly, several lines of evidence showed that the GS1 and AS isoforms are mainly located in the veins and more precisely in the phloem tissues (Bernard *et al.*, 2008; Lothier *et al.*, 2011; Ohashi *et al.*, 2015). It is therefore not surprising that compared with glutamate and aspartate, accumulation of glutamine and asparagine in the phloem-rich midvein is higher than in the laminae (Orsel *et al.*, 2014). The reason why glutamine and asparagine are more abundant in the young leaves than in the old ones is most probably related to the relative abundance of their glutamate and aspartate precursors. If precursors are more abundant, it seems logical that products are too. Interestingly, glutamine concentrations were remarkably high in the youngest leaves of HN plants as compared with those from LN plants. This can be explained by two mechanisms. Firstly, ammonium primary assimilation is very active in young leaves under HN conditions in particular. Secondly, cytosolic glutamine synthetases located in the veins are also closely involved in ammonium primary assimilation (Lothier *et al.*, 2011). Watanabe *et al.* (2013) proposed that the glutamine/glutamate and asparagine/aspartate ratios are appropriate to estimate the rate of interconversion and of nitrogen export. As such, these authors observed higher glutamine/glutamate and asparagine/aspartate ratios in the old leaves of Arabidopsis compared with the young leaves. In oilseed rape, we consistently found higher glutamine/glutamate and asparagine/aspartate ratios in the old laminae tissues. However, lower glutamine/glutamate and asparagine/aspartate ratios were observed in the old midveins. This can indicate that if the glutamate to glutamine and aspartate to asparagine conversions also occurred in the midveins, where the GS1 and AS isoforms are present, the export of glutamine and asparagine through the phloem to the sinks could lead to a higher decrease of midvein glutamine and asparagine contents relative to the glutamate and aspartate contents.

Proline metabolism is tightly regulated by the leaf d evelopmental stage (Masclaux *et al.*, 2000; Faës *et al.*, 2015). Surprisingly, in oilseed rape, proline decreased in the laminae with senescence and increased in parallel in their veins. This kind of ‘push-down, pop-up’ phenomenon could facilitate phloem loading in the old leaves, especially when nitrate is high. The fact that proline contributes to nitrogen remobilization and provides a nitrogen source for glutamine and asparagine synthesis in the veins of old leaves thus seems highly probable. Indeed, recent evidence showed that proline dehydrogenases, involved in the catabolism of proline, are expressed specifically in the veins (Funck *et al.*, 2010; Faës *et al.*, 2015).

As for glutamine and asparagine, the higher allocation of glucose and fructose in the midvein was prominent. In the laminae, the ranges of concentration of glucose, fructose, and sucrose were quite similar. In contrast, in the midvein of young leaves, the concentrations of fructose and glucose were 10–20 times higher than that of sucrose. In young leaves, they were also 10–20 times higher in the midvein compared with the laminae. While the leaf ageing effects in laminae were m oderate, a sharp hexose decrease was observed in the midvein with ageing. Such discrepancies between sucrose and hexoses and between midvein and laminae address the question of the role of the sucrose and hexose transporters located in the vein tissues. The SWEET facilitators are suspected to play a role in the lateral transfer of sugars from phloem to xylem and *vice versa* (Hölttä *et al.*, 2009; Jian *et al.*, 2016; Zakhartsev *et al.*, 2016). SWEET transporters consist of monomers, dimers, and oligomers, and can import or export hexoses and sucrose following their concentration gradients. The high amounts of glucose and fructose could play an important role in the source to sink transfer of sugars and could control allocation to the phloem or xylem saps. The relevance of using quantification of metabolites in the vein and petioles as a proxy of the phloem sap contents can then be considered. Recently, Lohaus and Schwerdtfeger (2014) compared the phloem sap composition with the whole leaf composition. They showed that sucrose was higher in the phloem sap, while glucose and fructose were inversely higher in the whole leaf tissue. Our results suggest exactly the opposite. The contribution of the vein tissues including xylem and phloem parenchyma cells could manage sucrose and hexose conversions in a way d ifferent from that in the laminae tissues.

One striking result of this report is that the TCA compounds did not change during leaf senescence in the same manner depending on the nitrate conditions. In the laminae, all the TCA compounds decreased with ageing, except fumarate and malate. They showed a biphasic mode under HN and fumarate increased with ageing under LN. In the midvein, all the TCA compounds decreased under HN while malate, fumarate, and succinate increased. The reason why these organic acids changed in different ways could be related to the anaplerotic pathways cited above. These pathways could fulfil the TCA cycle with succinate from the catabolism of GABA, for example, and create disequilibrium in the TCA fluxes. Another possibility could be the effect of senescence on photorespiration. The glycine, glycolate, and glycine/serine ratios indeed increased reliably in oilseed rape during laminae senescence, thus showing a decrease of the photorespiration activity. The increase of glyoxylate cycle and fatty acid β-oxidation in the glyoxysomes could also explain the increase of malate and fumarate during ageing (Chrobok *et al.*, 2016). Finally, the different patterns observed for fumarate and malate could be related to the fact that the TCA cycle is not cyclic, as shown by Tcherkez *et al.* (2009), and works in different ways d epending on whether it is the day or the night. It is highly possible that the respective contributions of the day and night metabolism are modified during leaf ageing. Indeed, several dark-induced or night-expressed genes are also senescence related (Fujiki *et al.*, 2001; Masclaux-Daubresse *et al.*, 2002, 2005). Finally, it is interesting to mention that the much higher concentrations of malate in the midvein compared with laminae in the nitrate-limited plants (Supplementary Fig. S4) are consistent with (i) the potential role of malate as a shoot to root signalling molecule for nitrate uptake (Touraine *et al.*, 1992) and (ii) the fact that malate is the predominant organic acid in the xylem and phloem saps (Ziegler, 1975).

As mentioned before, leaf senescence consists of cell d egradation, and at the same time many antioxidant and quality control mechanisms support cell longevity. The sharp increase in α-tocopherol and ascorbate during senescence in parallel with a dehydroascorbate decrease shows that antioxidants efficiently control oxidative stress throughout leaf development. The location of ascorbate and dehydroascorbate in veins and laminae of oilseed rape indicated that ascorbate concentrations were higher in veins compared with laminae, while dehydroascorbate concentrations were higher in laminae than in veins. Oxidative regulation appears more efficient in oilseed rape veins where the chloroplast number is more limited than in the green laminae tissues where reactive oxygen species formation increases in the chloroplasts during ageing (Suzuki *et al.*, 2012). This interpretation is consistent with the fact that α-tocopherol is mostly exclusively present in the laminae.

Regarding the strong effects of leaf ageing and senescence on the relative metabolite concentrations in veins and laminae, our study suggests that the main checkpoint for source to sink nutrient remobilization could be located in the veins and could consist of metabolite interconversions. The fact that several amino acid and sugar transporters have been described in the literature to be differentially expressed d uring leaf senescence suggests that phloem or xylem loading could also be a limiting point (Masclaux-Daubresse *et al.*, 2008; Seo *et al.*, 2011). For that reason, studying senescence programmes in laminae and veins separately could reveal new and important cues on the source–sink relationships playing a role in nutrient remobilization during leaf senescence.

In conclusion, studying metabolic changes in veins during leaf senescence in more depth could reveal as yet unknown checkpoints in the control of nutrient source to sink management.

## Supplementary data

Supplementary data are available at *JXB* online.

Data Set S1. Annotated data matrix.

Data Set S2. Hierarchical clustering analysis of metabolites significant by one-way ANOVA .

Data Set S3. Metabolite concentrations in oilseed rape laminae and midvein.

Fig. S1. Expression of *BnaGLN1* (*GS1*) and *BnaGLS* (*GS2*) genes in *B. napus* laminae.

Fig. S2. PCA analysis.

Fig. S3. Senescence effects in laminae and midvein under high and low nitrate conditions.

Fig. S4. Metabolite relative abundance in the midvein relative to laminae.

## Supplementary Material

supplementary_dataset_S1

supplementary_dataset_S2

supplementary_dataset_S3

supplementary_figures_S1_S4
