# Supplementary material for: Metabolomics of laminae and midvein during leaf senescence and source–sink metabolite management in Brassica napus L. leaves
Source: J Exp Bot. 2017 Sep 6;69(4):891–903. doi: 10.1093/jxb/erx253 (PMC5853214; doi:10.1093/jxb/erx253)

Metabolomics of laminae and midvein during leaf senescence and source-sink metabolite management in *Brassica napus* L. leaves

Gilles Clément[1a], Michaël Moison[1a], Fabienne Soulay[1], Michèle Reisdorf-Cren[1] and Céline Masclaux-Daubresse[1*]

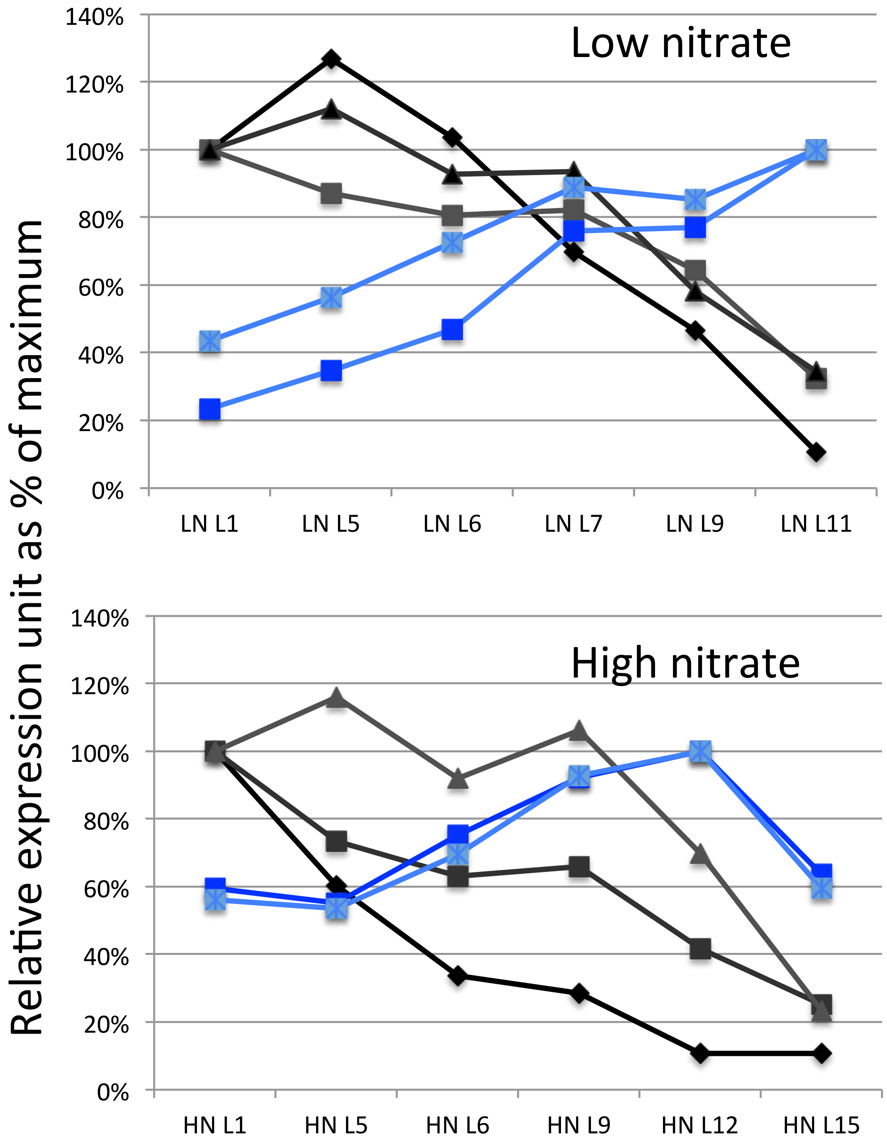

Supplemental Figure S1: Expression of BnaGLN1 (GS1) and BnaGLS (GS2) genes in *B. napus* laminae depending on nitrate availability and leaf ageing. Expression of genes was measured by Orsel et al. (2014) using QRT-PCR. Decrease of BnaGLN1 gene expressions and increase of BnaGLN2 gene expressions in young leaves relative to old leaves show the senescence gradation in the six leaf ranks chosen for our study. GS1 genes: *BnaA.GLN1.1a* (black losange); *BnaC.GLN1.1a* (black square); *Bna1.GLN1.4a* (black triangle). GS2 genes: *BnaGLS1* (dark blue square); *BnaGLS2* (light blue square).

Supplemental figure 2: PC1/PC2 axis

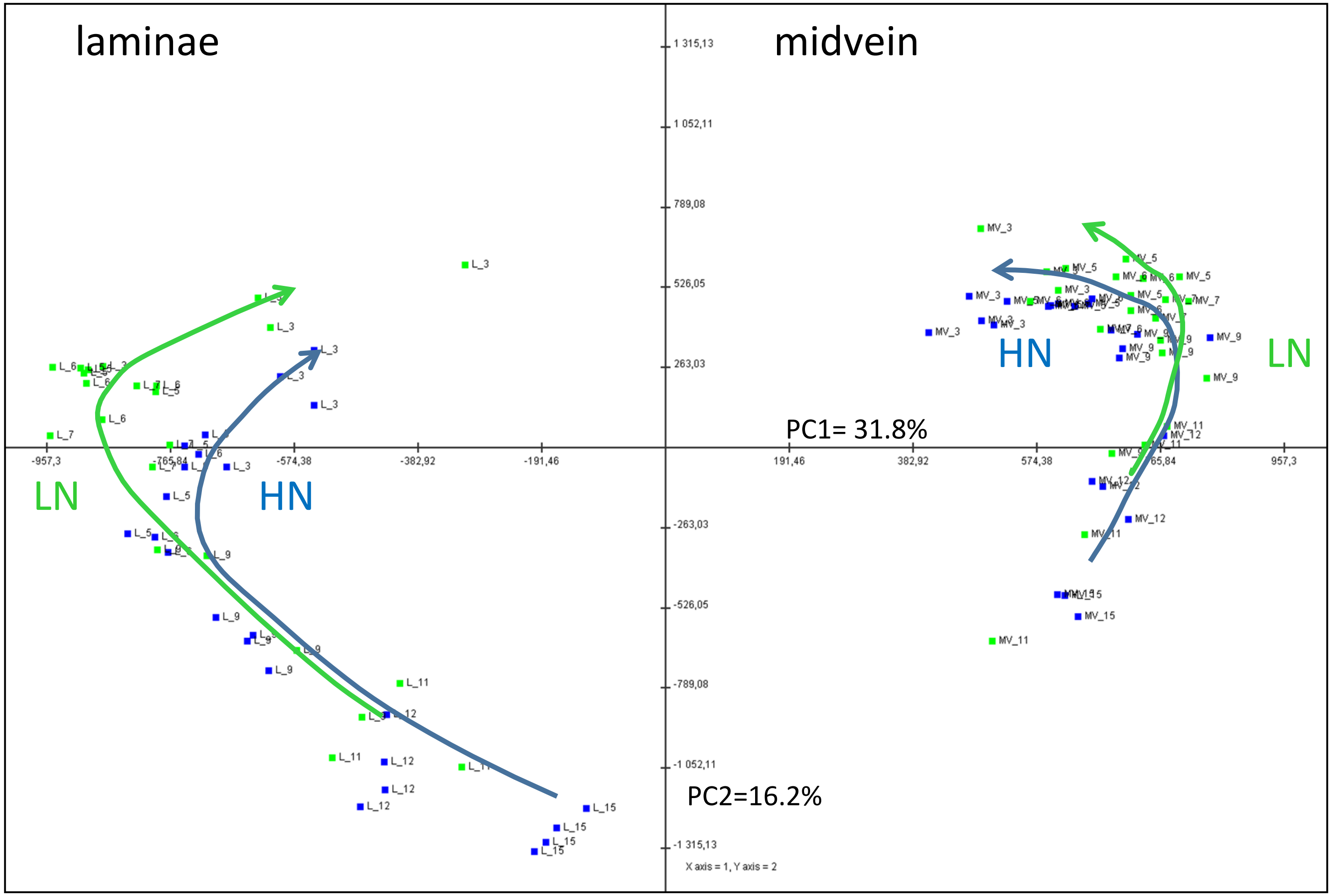

Supplemental figure 2 continue: PC1/PC3 axis

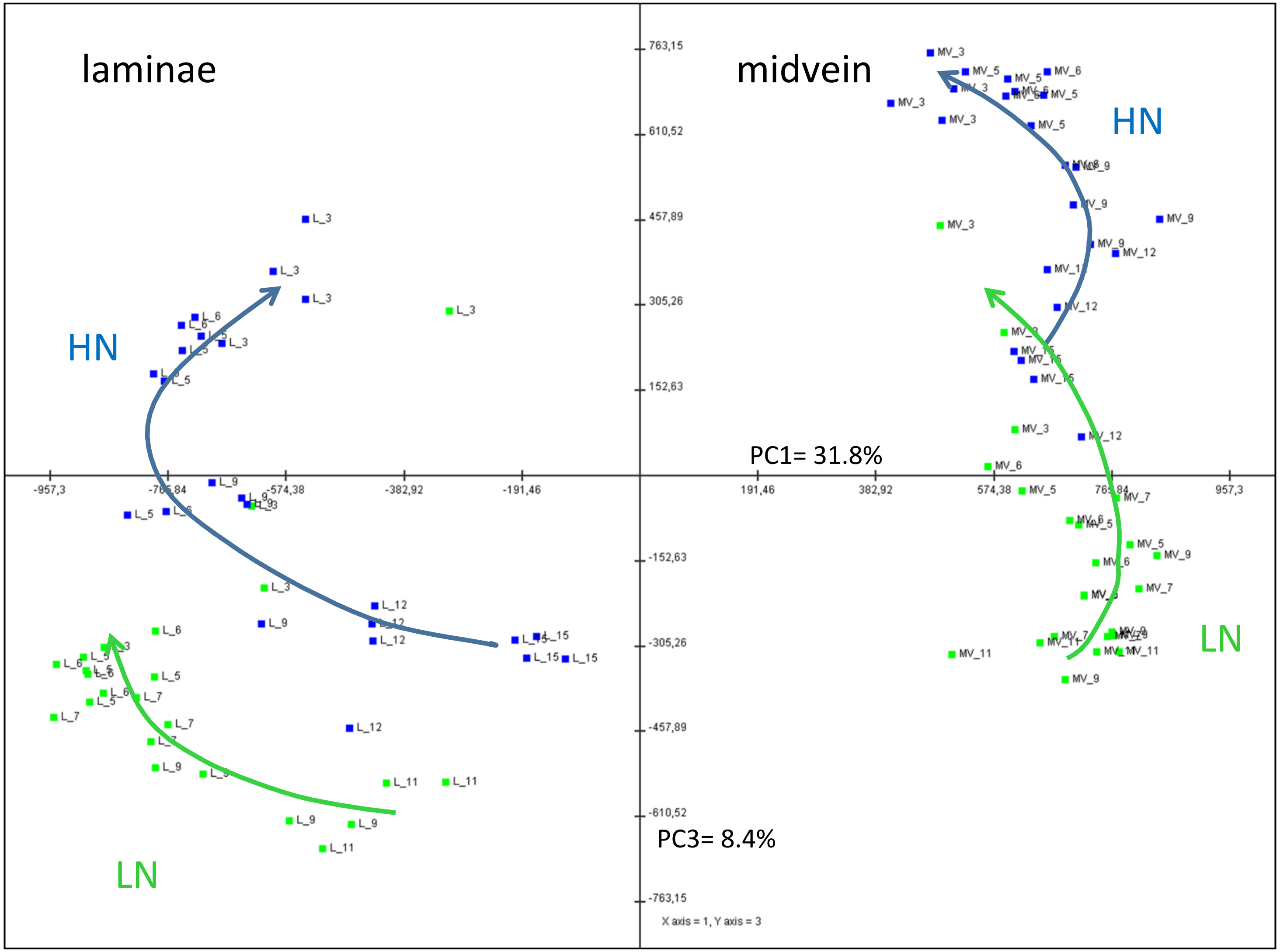

Supplementary figure 3: Senescence effects in laminae and veins under high and low nitrate conditions.

Data are nomalized to the youngest leaves of plants grown under the same nutrient condition (L11 for Low N and L15 for High N).

Log2 ratio are shown.

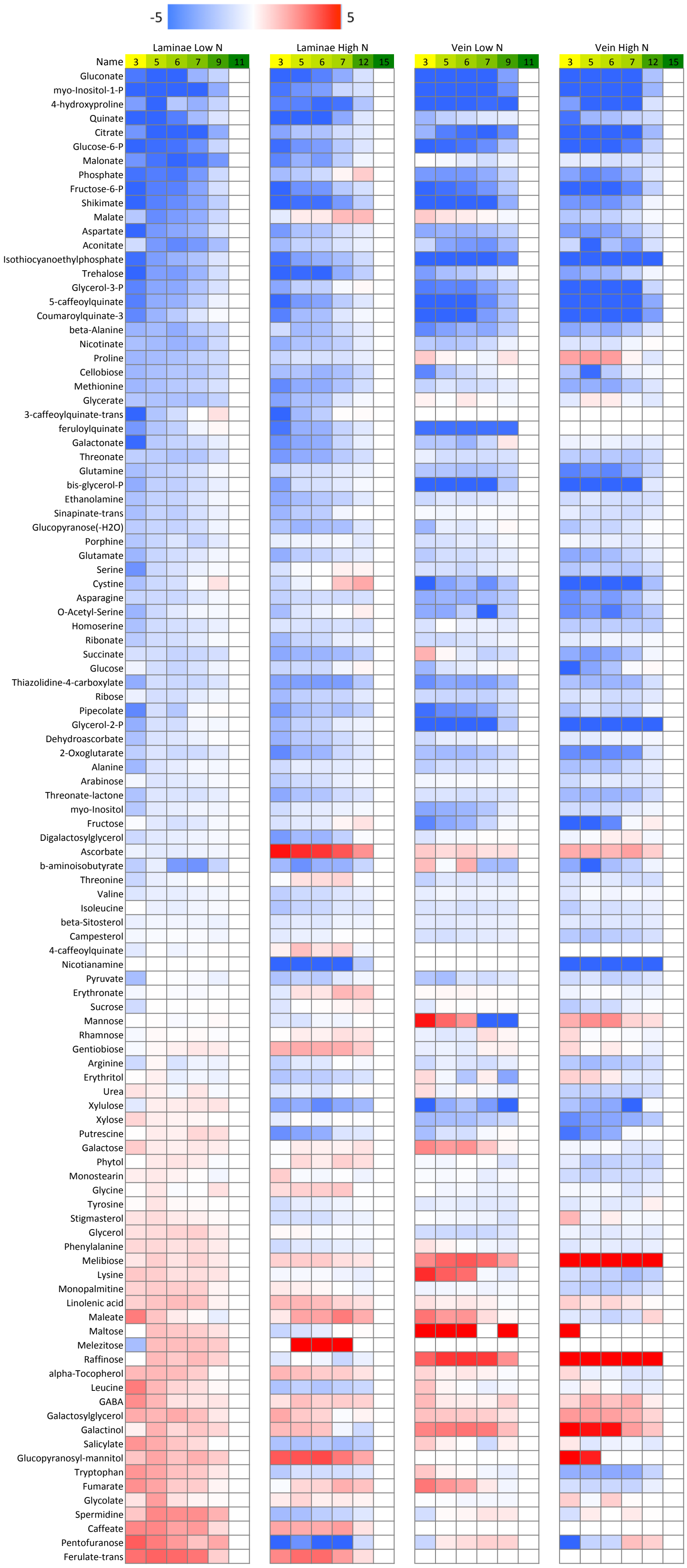

Supplemental figure 4: Abundance in veins relative to laminae.

Heat map representing the magnitude of the relative abundance of metabolites in the vein compared to the laminae of oilseed ra
Log2 (concentration in vein/concentration in laminae)

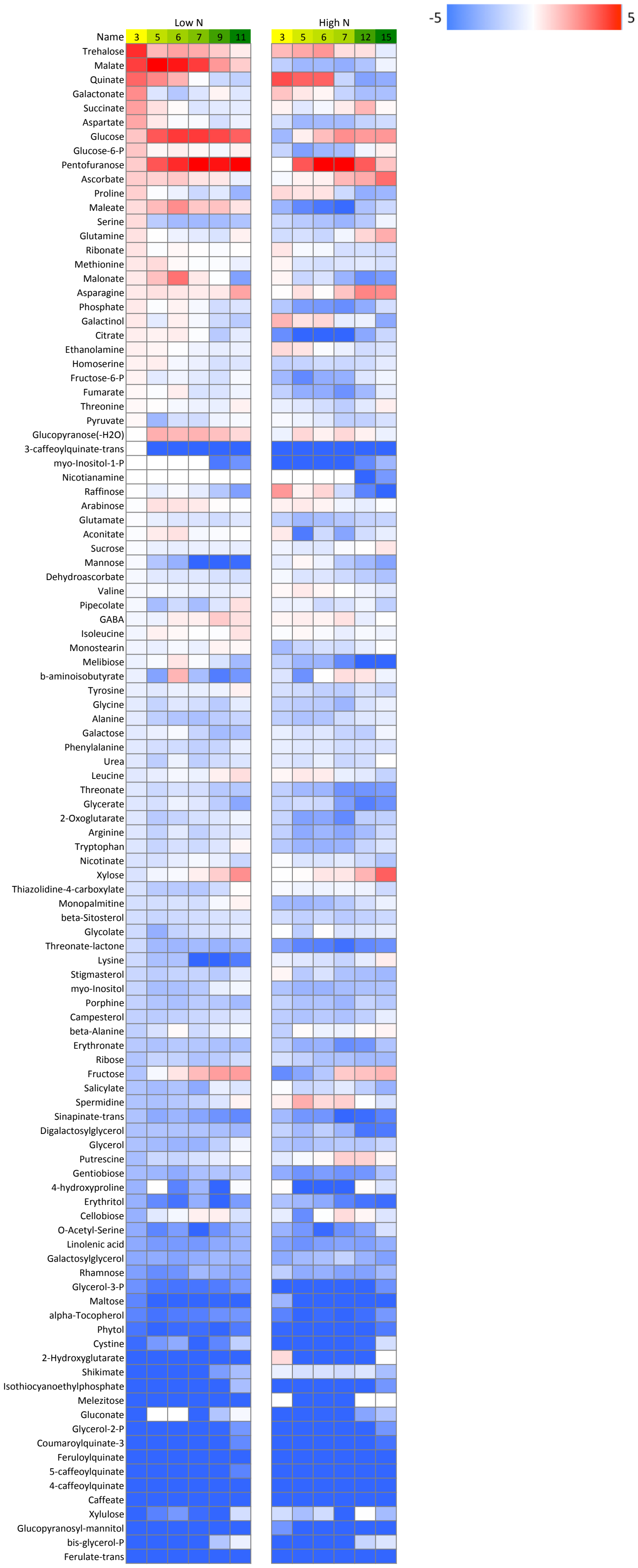

Supplement: supplementary_figures_S1_S4 [file erx253_suppl_supplementary_figures_s1_s4.pdf]
